# Electrospun Zein/Gelatin Scaffold-Enhanced Cell Attachment and Growth of Human Periodontal Ligament Stem Cells

**DOI:** 10.3390/ma10101168

**Published:** 2017-10-12

**Authors:** Fanqiao Yang, Yingling Miao, Yan Wang, Li-Ming Zhang, Xuefeng Lin

**Affiliations:** 1 Guanghua School of Stomatology, Sun Yat-sen University, Guangdong Provincial Key Laboratory of Stomatology, Guangzhou 510080, China; yangfanq@mail2.sysu.edu.cn (F.Y.); wang93@mail.sysu.edu.cn (Y.W.); 2 School of Materials Science and Engineering, Sun Yat-sen University, Guangzhou 510275, China; tpfachristina@163.com

**Keywords:** zein, gelatin, electrospun, human periodontal ligament stem cells, cytocompatibility, tissue engineering

## Abstract

Periodontitis is a widespread dental disease affecting 10 to 15% of worldwide adult population, yet the current treatments are far from satisfactory. The human periodontal ligament stem cell is a promising potential seed cell population type in cell-based therapy and tissue regeneration, which require appropriate scaffold to provide a mimic extracellular matrix. Zein, a native protein derived from corn, has an excellent biodegradability, and therefore becomes a hotspot on research and application in the field of biomaterials. However, the high hydrophobicity of zein is unfavorable for cell adhesion and thus greatly limits its use. In this study, we fabricate co-electrospun zein/gelatin fiber scaffolds in order to take full advantages of the two natural materials and electrospun fiber structure. Zein and gelatin in four groups of different mass ratios (100:00, 100:20, 100:34, 100:50), and dissolved the mixtures in 1,1,1,3,3,3-hexafluoro-2-propanol, then produced membranes by electrospinning. The results showed that the scaffolds were smooth and homogeneous, as shown in scanning electron micrographs. The diameter of hybrid fibers was increased from 69 ± 22 nm to 950 ± 356 nm, with the proportion of gelatin increase. The cell affinity of zein/gelatin nanofibers was evaluated by using human periodontal ligament stem cells. The data showed that hydrophilicity and cytocompatibility of zein nanofibers were improved by blended gelatin. Taken together, our results indicated that the zein/gelatin co-electrospun fibers had sufficient mechanical properties, satisfied cytocompatibility, and can be utilized as biological scaffolds in the field of tissue regeneration.

## 1. Introduction

Periodontitis is a chronic inflammatory disorder that progressively destroys supporting tissues of the teeth, and ultimately leads to tooth loss through consequent irreversible alveolar bone damage. According to the reports from World Health Organization Global Oral Health Data Bank, advanced periodontal diseases with deep pocket depth (>6 mm) affect 10 to 15% of the worldwide adult population [1], yet the current treatments are far from satisfactory. Nowadays, periodontitis can be prevented and controlled by conventional therapies, namely, periodontal scaling and root planning, gingival curettage, and laser and photodynamic therapy [2]. However, these treatments are unable to promote tissue regeneration or repair the bone defect. This is why guided tissue regeneration (GTR) combined with bone graft materials has been adopted, though the clinical outcome is uncertain and far from satisfactory [3]. Periodontal tissue engineering has a bright prospects and is regarded as a fundamental strategy for periodontitis treatment [4], especially for alveolar bone regeneration. Human periodontal ligament stem cells (PDLSCs), a subpopulation with mesenchymal stem cells (MSCs), can be easily obtained from periodontal tissues, and the strong ability of formation of new bone, cementum, functional periodontal ligament and blood vessel has been proven [5,6,7]. Accumulating evidence implies human PDLSCs can act as a seed cell in cell-based therapy and tissue regeneration [8,9]. Recently, it has been reported that the bone formation was enhanced when combined PDLSCs with bovine-derived bone mineral materials, and the effect of clinical application on treating periodontal defects turned out to be desirable [10]. Scaffolds are key elements for delivering and retaining cells and molecules, mainly the constituents and geomorphology [11]. Electrospinning is the currently most popular way to produce three-dimensional porous structures with large surface-to-volume ratio, which helps cell migration and substance diffusion (nutrient, water, and oxygen), providing essential basis for cell growth [12].

Structural proteins are critical components of the extracellular matrix (ECM) that provides appropriate strength and toughness [13,14]. Moreover, two or more polymers are often blended to overcome the limitations [15]. Zein is an alcohol-soluble prolamin protein obtained from corn and was approved as a generally safe food-grade ingredient by the U.S. Food and Drug Administration [16]. Its natural biodegradability, good biocompatibility, and excellent electrospinnability allow zein-based biomaterials to become potential absorbable scaffolds for tissue regeneration [17,18,19]. The hydrophobicity of zein enables it to survive in gastric acid and resist enzymatic hydrolysis, leading to applications in encapsulation, drug controlled release systems (DCRS), and targeted delivery [20,21,22]. On the other hand, the strong hydrophobic property leads to poor cell affinity [19]. Gelatin, a fibrous protein extracted from denatured native collagen (a major component of the ECM), shares a similar structure with native collagen. With good biocompatibility and low immunogenicity, gelatin has been extensively investigated in regenerating tissues such as synthetic polymer polycaprolactone, poly(lactic-co-glycolic acid) or crosslinking with chondroitin sulfate [23,24,25,26]. However, the super hydrophilicity and solubility of gelatin in turn bring high dissolubility [27].

This article is aimed to fabricate zein/gelatin fiber scaffolds by co-electrospinning technique and evaluate cell attachment and growth of human PDLSCs cultured on zein/gelatin fiber scaffolds. Therefore, surface morphology, fiber diameter, mechanical performance, hydrophilicity, and degradability of the co-electropinning zein/gelatin scaffolds were characterized. The cytocompatibility of zein/gelatin membrane was evaluated by in vitro cell attachment and proliferation studies. The results of this study may provide some useful information on appropriate dental scaffolds selection and fabrication, in order to achieve bone regeneration in the recovery of periodontitis.

## 2. Results

### 2.1. Characterization of Zein/Gelatin Nanofibrous Matrix

**Figure 1:**
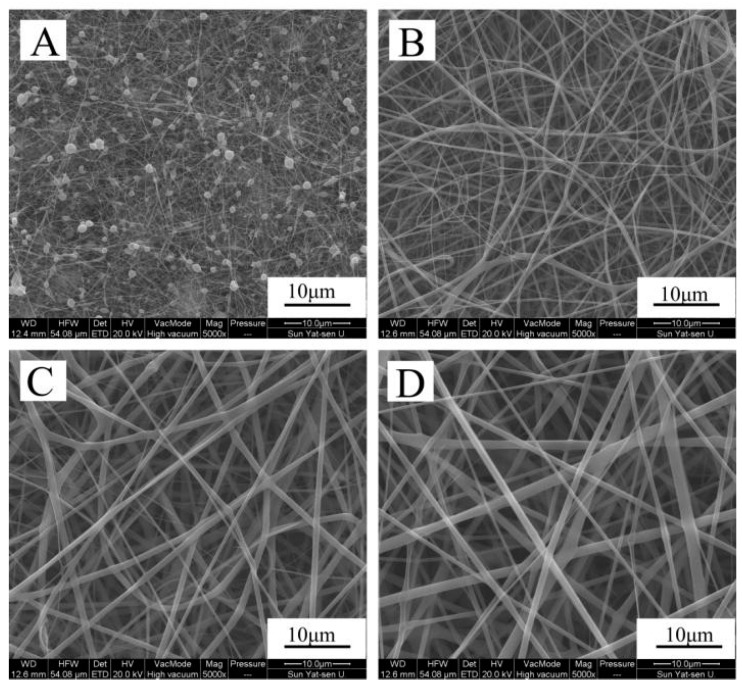
Scanning electron microscope (SEM) images of the surface morphology of zein (**A**); zein/gelatin-1 (**B**); zein/gelatin-2 (**C**); and zein/gelatin-3 (**D**) electrospun nanofibers.

**Figure 2:**
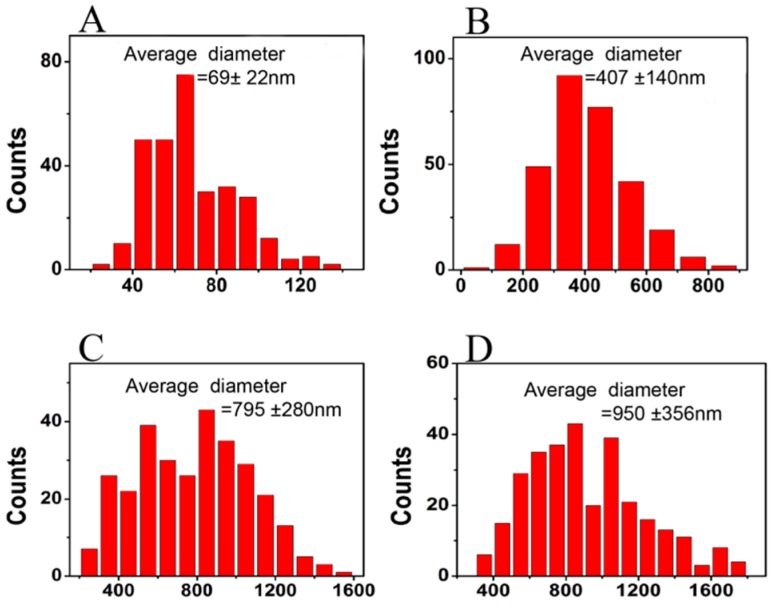
The fiber diameter distribution of zein (**A**); zein/gelatin-1 (**B**); zein/gelatin-2 (**C**); and zein/gelatin-3 (**D**) electrospun nanofibers.

The scanning electron microscope (SEM) pictures in Figure 1 displays the morphology of pure zein, zein/gelatin-1, zein/gelatin-2, and zein/gelatin-3 electrospun nanofibers. In Figure 1A, many large beads in the pure zein membrane could be observed. However, all the zein/gelatin nanofibers in Figure 1 were fabricated with interconnected pores and a large surface area as well as randomly distributed smooth ultrafine fibers under the same electrospinning conditions. It was clearly seen that when the mass ratio of zein to gelatin increased from 100:00 to 100:20, and eventually 100:50, the beads disappeared in the zein/gelatin nanofibrous matrix. Both proteins dissolved well in 1,1,1,3,3,3-hexafluoro-2-propanol (HFIP), even when the ratio of gelatin to zein was up to 50%. However, the blended solution presents difficulty in fiber formation when keep adding gelatin to zein. Figure 2 presents the fiber diameter distribution according to 300 randomly chosen fibers from the SEM images. Pure zein showed thinner fibers with a small average diameter of 69 ± 22 nm and a narrower fiber distribution. In contrast, after the addition of gelatin, the average diameter of zein/gelatin-1, zein/gelatin-2, and zein/gelatin-3 electrospun nanofibers increased visibly, exhibiting thicker fibers and a broader fiber distribution with a bigger average diameter of 407 ± 140 nm, 795 ± 280 nm, and 950 ± 356 nm, respectively.

The tensile test data of the zein/gelatin membranes are summarized in Table 1. For pure zein, the tensile strength and elastic modulus is 0.79 ± 0.12 MPa and 35.31 ± 5.53 MPa, respectively. After blending with gelatin, the tensile strength and elastic modulus increased noticeably; zein/gelatin-1 reached 1.32 ± 0.29 MPa and 63.18 ± 9.04 MPa; and zein/gelatin-2 reached 1.79 ± 0.16 MPa and 83.43 ± 5.83 MPa, respectively. Zein/gelatin-3, with 1.88 ± 0.22 MPa and 133.34 ± 11.22 MPa, respectively, showed a 138% raise in tensile strength and 278% raise of the elastic modulus when compared to pure zein.

**Figure 3:**
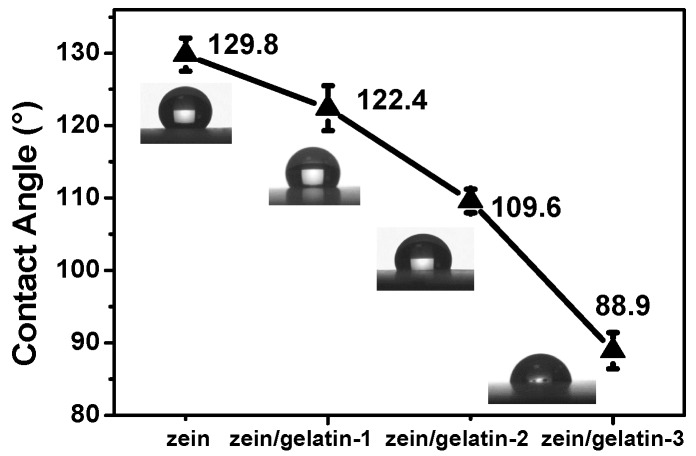
Contact angle pictures of zein, zein/gelatin-1, zein/gelatin-2, and zein/gelatin-3 electrospun nanofibers.

Figure 3 shows the contact angles of different nanofibrous matrices. Pure zein exhibited high hydrophobicity with a contact angle of 129.8 ± 2.3°. The contact angles of zein/gelatin-1, zein/gelatin-2, and zein/gelatin-3 nanofibrous membranes were 122.4 ± 3.1°, 109.6 ± 1.6°, and 88.9 ± 2.5°, respectively. As the gelatin content increased from 100:00 to 100:50, the water contact angle decreased from 129.8° to 88.9°. This result demonstrates that blending with gelatin could alter the surface wettability of zein from hydrophobic (contact angle over 90°) to hydrophilic (contact angle below 90°).

**Figure 4:**
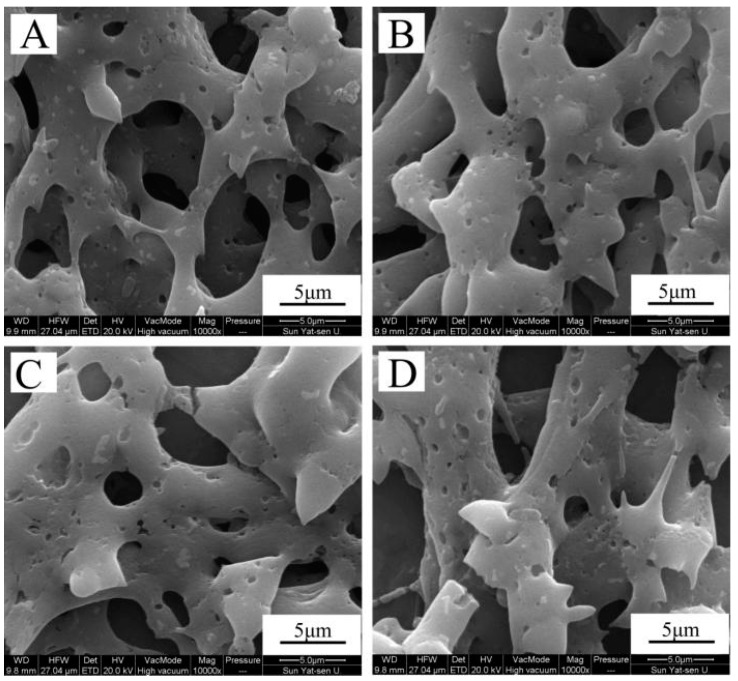
Morphological changes in zein (**A**); zein/gelatin-1 (**B**); zein/gelatin-2 (**C**); and zein/gelatin-3 (**D**) electrospun nanofibers after enzymatic degradation for 7 days.

The in vitro degradation of the nanofibrous matrix was measured using SEM. The morphological changes in the zein, zein/gelatin-1, zein/gelatin-2, and zein/gelatin-3 nanofibers from enzymatic degradation at 37 °C are shown in Figure 4. The broken area that appear on the fibers indicate that these scaffolds have been decomposed.

### 2.2. Characterization of Human PDLSCs

**Figure 5:**
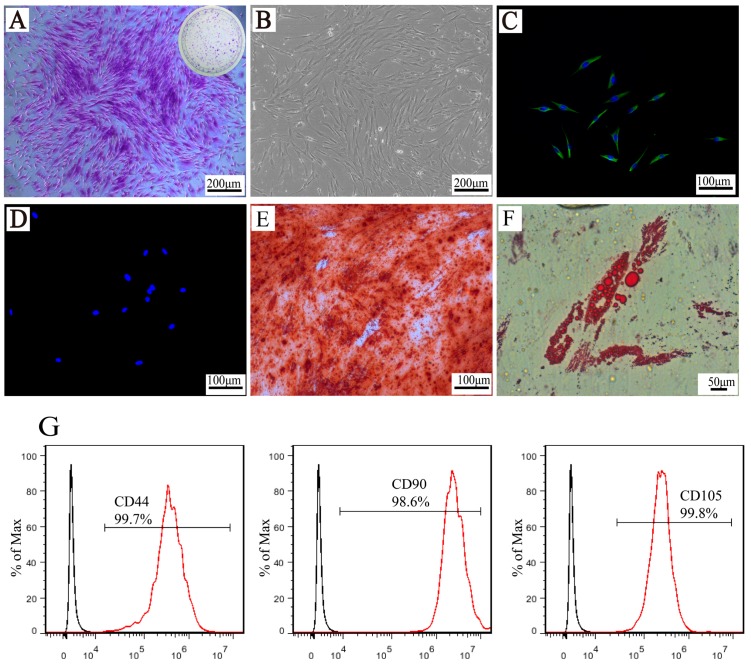

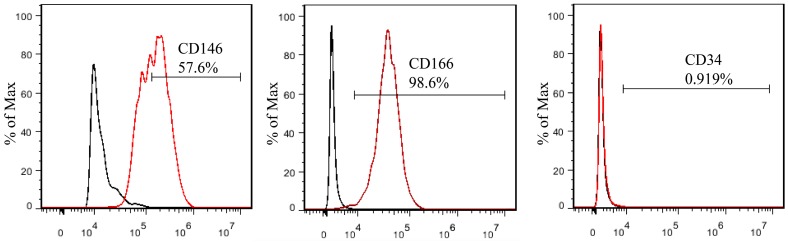
Characterization of human periodontal ligament stem cells (PDLSCs). Single-cell colony formation (**A**); and cell adhesion and proliferation on a tissue culture plate (**B**); (**C**,**D**) Show vimentin/nuclei and cytokeratin/nuclei staining; (**E**) Osteogenic differentiation (alizarin red staining) and (**F**) adipogenic differentiation (oil red O staining). Immunophenotype expression results from flow cytometric analysis (**G**).

After 3–5 days primary culture, the cells successfully migrated from the tissue blocks and adhered to the culture plate. Figure 5A shows one colongenic cluster of passage 2 cells, which reveals its excellent self-renewal and proliferative ability. Then, the colony-forming cells were collected and expanded, and the human PDLSCs displayed the characteristic spindle shape shown in Figure 5B. The immunofluorescence staining presented vimentin(+) (Figure 5C) and cytokeratin(−) (Figure 5D). As for the multipotential differentiation ability of human PDLSCs, mineralized nodules were clearly observed after 28 days of osteogenic induction and lipid droplets formed after 21 days of adipogenic induction, which provided strong evidence of the excellent osteogenic/adipogenic differentiation ability of human PDLSCs. From the flow cytometric analysis shown in Figure 5G, the adherent cells highly expressed MSC markers, including CD44 (99.7%), CD90 (98.6%), CD105 (99.8%), CD146 (57.6%), and CD166 (98.6%), and negatively expressed the hematopoietic stem cell marker CD34 (0.919%). In conclusion, the colony-forming cells could well adhere to plastic, gradually displayed a fibroblast-like shape similar to that reported in the literature [28], positively expressed the mesenchymal marker vimentin, and did not express the epithelial marker cytokeratin [29]. The cell immunophenotypes of human PDLSCs expressed in vitro were consistent with the data of previously published studies [30,31,32]; namely, CD44, CD90, CD105, and CD166 were expressed above 95% and CD34 were expressed below 2%. Furthermore, these cells have pluripotency differentiation potential. Therefore, according to the literature [33], isolated human PDLSCs were indeed successfully obtained.

### 2.3. Cytocompatibility

**Figure 6:**
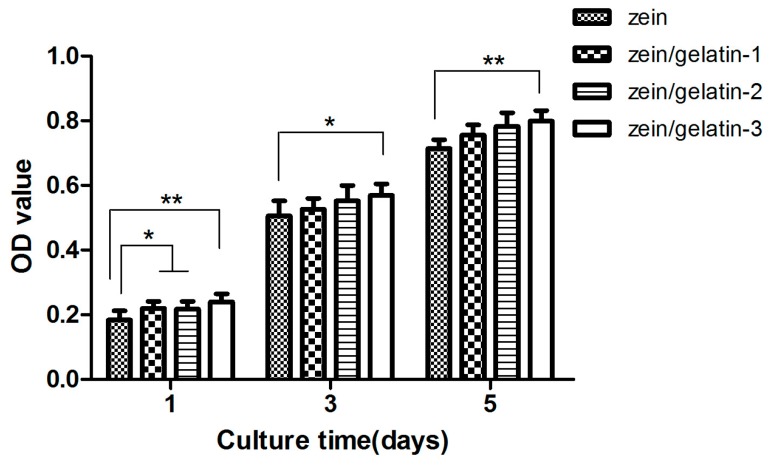
Characterization of cell proliferation on electrospun membrane containing zein with different gelatin ratios at different times. (*), and (**) represent a statistical significance of *p* < 0.05 and *p* < 0.01, respectively.

The characteristics of the scaffold matrix, such as surface morphology, surface wettability, and degradation property, strongly affect cell attachment and growth. An MTS (CellTiter 96^®^ AQueous One Solution Cell Proliferation Assay, MTS, Promega Corporation, Madison, WI, USA) assay was used to evaluate the toxicity of the scaffolds. Figure 6 demonstrates the proliferation of human PDLSCs seeded on the scaffolds at different function times. Compared to that of pure zein, the cell proliferation raised along with increasing amount of gelatin and longer culture time. On day 1, zein/gelatin-1 and zein/gelatin-2 showed greater cell proliferation rate than zein (*p* < 0.05). Furthermore, a statistically significant difference was observed on the first day and the fifth day between zein and zein/gelatin-3 (*p* < 0.01). Thus, these results indicate that the electrospun zein/gelatin nanofibrous scaffolds favor cell adhesion and proliferation when they were used as scaffolds for human PDLSC culture.

**Figure 7:**
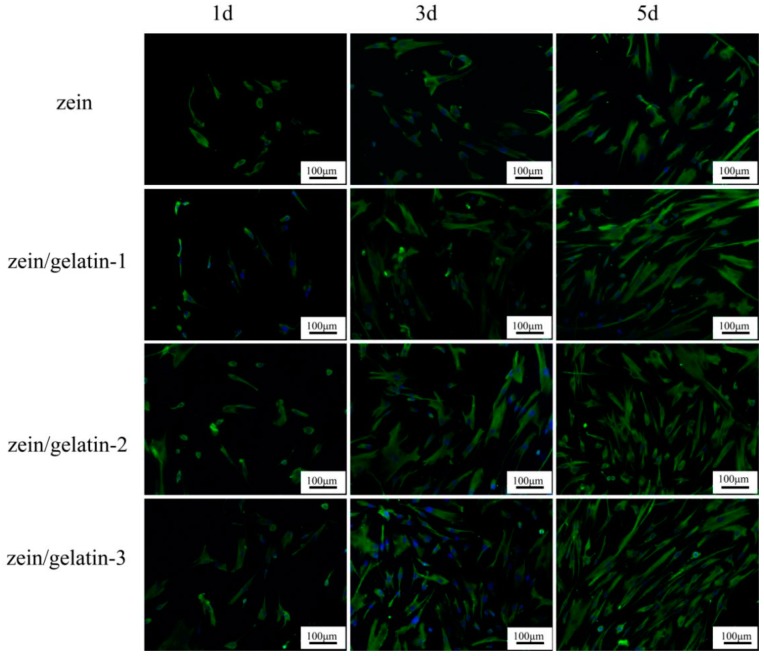
Vimentin and nuclei staining of human PDLSCs on pure zein, zein/gelatin-1, zein/gelatin-2, and zein/gelatin-3 nanofibers on different days.

On days 1, 3, and 5, cell attachment on the electrospun scaffolds are observed in Figure 7. Initially, cells in each group exhibited a round morphology on pure zein, but with the gradual addition of the gelatin component, the cell number increased slightly. The cells were gradually elongated to the spindle shape with longer culture time, which is especially apparent in zein/gelatin-3. Besides, the results showed that the cell numbers on the different blended fiber scaffolds were consistent with the MTS assay. Apparently, zein/gelatin-3 exhibited the highest proliferation ability at different function times.

**Figure 8:**
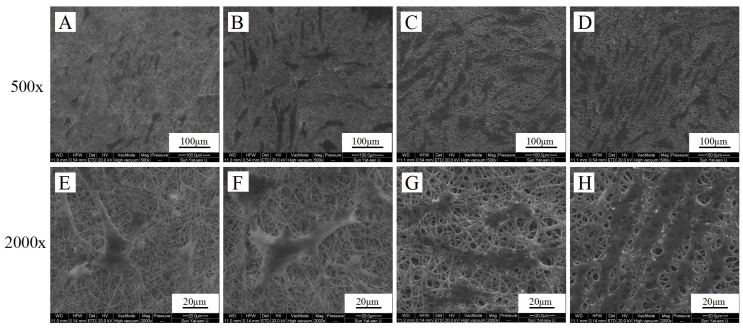
Morphology of human PDLSCs cultured on pure zein (**A**,**E**); zein/gelatin-1 (**B**,**F**); zein/gelatin-2 (**C**,**G**); and zein/gelatin-3 (**D**,**H**) for 6 days at different magnifications.

SEM images of different magnifications showing human PDLSCs growing on blended zein/gelatin membranes were displayed in Figure 8. The majority of the human PDLSCs maintained the fibroblast morphology and were well spread on all sample surfaces. The zein/gelatin-3 sample had the best adhesion and spreading of human PDLSCs; meanwhile, all these fibrous structures displayed a relatively stable state in the medium for 6 days.

**Figure 9:**
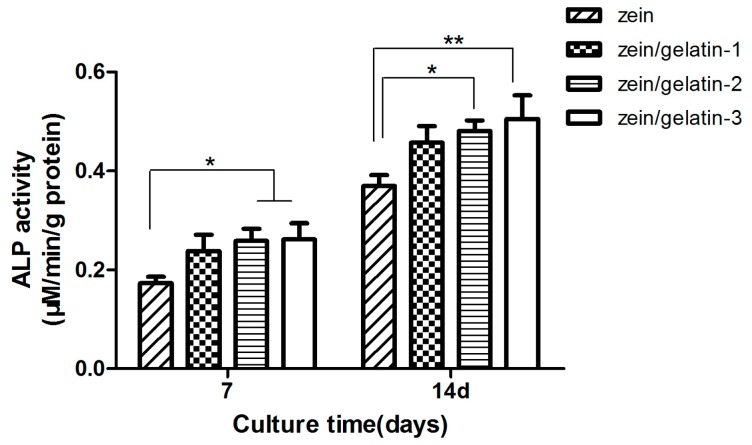
Alkaline phosphatase (ALP) activity of human PDLSCs after 7 and 14 days culture on zein, zein/gelatin-1, zein/gelatin-2, and zein/gelatin-3 nanofibrous membranes. (*), and (**) represent a statistical significance of *p* < 0.05 and *p* < 0.01, respectively.

The zein/gelatin membrane groups, zein/gelatin-2 and zein/gelatin-3 showed noticeably higher alkaline phosphatase (ALP) activity compared to the zein group displayed in Figure 9. The results indicate that the zein/gelatin membrane provided a more beneficial environment for human PDLSC differentiation.

## 3. Discussion

Nanofiber membranes prepared by electrospinning have attracted much attention owing to their outstanding bionic structure of native ECM to provide cells with a mimic growing environment. However, large beads could be seen in the pure zein group [34]. Interestingly, it is observed that there was no bead formation, as the concentration (w/v) of zein/HFIP) increased from 10 to 20% [35]. We speculate that the formation of beads might attribute to low concentration and viscosity of zein in HFIP. We failed to add gelatin to fabricate electrospun membranes when the proportion of zein/HFIP was raised up to 20% because of the high viscosity, and this is why we chose 10% for zein/HFIP in this study. It is reported that along with the content of gelatin enhancing, the fiber diameter increased [36]. We found that a higher proportion of gelatin generates thicker membrane, and this is similar with the results of Hu's group [37]. Besides, as viscosity increases with gelatin, a larger fiber diameter is achieved, but with the constant addition of gelatin, the system viscosity becomes so high that it causes difficulties in fiber formation. In our opinion, surface morphology and diameter distribution of the nanofibers could be adjusted to acquire better similarity to the natural ECM by changing the system viscosity using different gelatin and zein ratios.

Mechanical performance of the scaffolds is an important parameter in cell culture and tissue engineering. Tensile strength reflects the fracture resistance of the material. The gelatin hybrid group displayed higher tension resistance compared to pure zein, which may be due to the enhanced chain entanglements in the blended zein/gelatin fiber scaffolds with higher viscosity in HFIP [19], whereas the strain at break decreased slightly. Our results showed the addition of gelatin remarkably increased the elastic modulus of nanofibrous mats. It is indicated that appropriate amount of gelatin could improve the stress of zein and too much gelatin could lead to a strain decrease and consequent brittle membranes.

Fiber diameters of bio-scaffolds plays an important role in cell growth, attachment, infiltration, and differentiation by changing the scaffolds characteristics such as scaffold pore size, mechanical property and surface tomography [38]. Since fiber diameter is the dominate factor controlling pore size, increased fiber diameter may lead to larger pore size [39,40]. The difference of pore size can greatly affect cell behavior and it is found that human dermal fibroblasts on small pore size scaffold showed delayed cell response [41]. However, the cell behavior cannot be decided by fiber diameter alone, since it is also influenced by initial cell seeding density, cell phenotypes, and constituents of biomaterials [42]. In our study, four different fiber diameters of electrospun zein/gelatin fiber scaffold was prepared and tested. The results showed that with larger fiber diameter, the cell attachment and proliferation rate of human PDLSCs were enhanced. This may be due to larger pore size or other mechanical property change, which will be studied in our future research.

The surface wettability greatly influences cell attachment behaviors and can be determined by water contact angle measurement [43]. There are reports that the hydrophilicity of electrospun membranes could be altered by blending with natural polymer [37,44]. In our study, zein/gelatin-3 had the best surface wettability and showed the best cell affinity among the various mixing ratio of zein and gelatin. It is interesting to note that biodegradability of the zein/gelatin membranes allow themselves to gradually disappear during the regeneration process (Figure 4), which means the patients won’t need another operation to remove the scaffold after the implantation of the materials. The α-helix and β-sheet structure of zein helps the electrospun fibrous membrane keep undegraded on the early stage [45], and together with our data, we hypothesize that when the scaffold is used for periodontal defect restoration in vivo, it can stay stable and functional, and will gradually diminish in the long term.

Protein-based biomaterials are favorable for cell adhesion because the protein layer on their surface can create interactions between cells and biomaterials [46]. In our study, pure zein membranes presented lower cell adhesion than the zein/gelatin groups. The improvement achieved by gelatin addition is because of the substantial integrin binding sites and excellent hydrophilicity [36,47]. Recent reports demonstrated that the increase of gelatin content enhances the attachment and proliferation of L929 mouse fibroblast cells [48], and revealed that cell responses may be closely related to the amino and carboxyl groups of gelatin [49]. With the co-electrospun technique, the porous surface morphology, mechanical properties, surface wettability, and cytocompatibility could be adjusted to a desired state by varying the ratios of zein and gelatin. The hybrid co-electrospun zein/gelatin fibrous membrane can work as a future bio-scaffold in restoration of periodontal tissue defect, especially the bone defect caused by periodontitis.

## 4. Materials and Methods

### 4.1. Materials

Zein (Z3625), gelatin from porcine skin (gel strength = 300 g Bloom, Type A), and collagenase type Ι were purchased from Sigma-Aldrich (St. Louis, MO, USA). 1,1,1,3,3,3-hexafluoro-2-propanol (HFIP) (99%) was purchased from Energy Chemical Company (Jiangsu, China). Standard culture medium (alpha-minimum essential medium with 10% fetal bovine serum, 1% penicillin, 1% streptomycin and 5 mM glutamine), dispase, and glutamine were bought from the Gibco Company (Grand Island, NY, USA), penicillin and streptomycin were purchased from the Hyclone Company (South Logan, UT, USA). The osteogenic medium consisted of standard culture medium supplemented with 100 μM L-ascorbic-2-phosphate, 20 μM dexamethasone, and 2 M β-glycerophosphate bought from Sigma-Aldrich. Adipogenic medium A (standard culture medium supplemented with 1 μM hydrocortisone, 10 μg/mL insulin, 0.5 mM 1-methyl-3-isobutylxanthine (IBMX) and 100 μM indomethacin) were purchased from Cyagen Biosciences Inc (Sunnyvale, CA, USA). 

### 4.2. Preparation of the Nanofibrous Membranes

Zein with a mass of 0.5 g was dissolved in HFIP to obtain a 10% (w/v) concentration and varying amounts of gelatin were added while stirring. Solutions of pure zein and three different ratios of zein/gelatin (100:20, 100:34 and 100:50) were prepared and named. For electrospinning, each blended solution was placed in a 5 mL glass syringe and the tip of the stainless steel needle was adjusted to the surface of the aluminum foil at a distance of 15 cm. Electrospun nanofibrous matrices of zein, zein/gelatin-1, zein/gelatin-2 and zein/gelatin-3 were prepared by applying a voltage of 20 kV with a solution flow rate of 0.6 mL/h. The electrospinning procedures for the pure zein and blended zein/gelatin membranes were performed under the same conditions.

### 4.3. Characterization of the Nanofibrous Matrix

The surface morphology of the electrospun nanofibrous matrix was observed by scanning electron microscope (SEM, JSM-6330F, JEOL, Tokyo, Japan). After the samples were sputter-coated with gold, they were observed using SEM with an accelerating voltage of 20 kV. The diameters of the nanofibers were measured using Image plus 6. Eventually, 300 random measurements were performed to get the average diameters value.

The mechanics performance was examined using an electronic universal testing machine (CMT6103, Sans, Shenzhen, China). The nanofibrous matrix was cut into a size of 10 × 30 mm^2^. After that, the measurement was completed with a stretching speed of 2 mm/min at 25 °C and relative humidity 46%. Three specimens of each sample were measured to display the average value.

The surface wettability of the nanofibrous matrices was measured with deionized water by using a contact angle meter (KRUSS DSA10-MK, KRUSS, Hamburg, Germany). Deionized water dropped automatically to the surface of the matrix and the micrographs were captured after 2 s. Three specimens of each sample were measured to display the average value.

The degradation ability of the electrospun nanofibers was evaluated by an enzymolysis test. The zein, zein/gelatin-1, zein/gelatin-2, and zein/gelatin-3 nanofibrous matrices were cut into 10 × 10 mm^2^ samples and immersed in a phosphate buffer saline (PBS) solution supplemented with collagenase type Ι (10 mg/mL). The matrices were taken out after 7 days and washed with deionized water three times. After vacuum drying, their morphologies were observed by SEM.

### 4.4. Cell Isolation and Culture

This study was approved by ethical committee of the Affiliated Stomatological Hospital of Sun Yat-sen University. Periodontal ligament tissues obtained from the non-diseased premolars were extracted owing to orthodontic demands at the Affiliated Stomatological Hospital of Sun Yat-sen University. The middle section tissues of the root surfaces were scraped, collected and subsequently digested with collagenase type I (3 mg/mL) and dispase (4 mg/mL) in a 37 °C, 5% CO^2^ incubator for 1 h. The tissues were cultured in standard culture medium. After cells migrated from tissues and reached 80% confluent, cells were seeded in a 10-cm diameter dish with a density of 900 cells. Twelve days later, these cells were fixed with 4% paraformaldehyde and subsequently stained with 1% crystal violet. Using microscope observations, cells number higher than 50 were characterized as a colony. Images were captured with a fluorescence microscope. Colony-forming cells were collected and passage 3–5 cells were used in the experiment described below.

Immunofluorescence staining was used to verify the origin of cells. Briefly, at passage 3, 2000 cells/well were seeded in 24 well tissue culture plates. Two days later, 4% paraformaldehyde was used to fix the cells. After that, the cells were permeabilized 10 min using 0.25% Triton X-100 and then incubated overnight with primary antibodies raised against cytokeratin and vimentin (1:200, Abcam, Cambridge, MA, USA). Subsequently, secondary antibodies (1:300, Abcam) were used for the cells and incubated for 45 min. After washing with PBS three times, the cells were incubated with Hochest 33342 (Sigma, Taufkirchen, Germany) for 2–3 min. Images were captured with the fluorescence microscope Zeiss Axio Observer Z1 (Carl Zeiss, Oberkochen, Germany).

Flow cytometry was applied to characterize the immunophenotype of the human PDLSCs. Cells at passage 3 were collected by a series of washing and resuspending processes. For each staining, the cells were incubated with anti-CD34, anti-44, anti-90, anti-CD105, anti-CD146 (phycoerythrin -labeled; 1:10; Becton Dickinson Biosciences, San Jose, CA, USA), and anti-CD166 (fluorescein isothiocyanate -labeled; 1:10; Life Technologies Corp., Carlsbad, CA, USA) on ice and away from light for 1 h. Subsequently, the cells were resuspended in 300 μL PBS. The immunophenotype staining was evaluated using a machine named BD Accuri C6 (Becton Dickinson Biosciences) and analyzed by CF Low Plus Software (Becton Dickinson Biosciences).

Osteogenic/adipogenic assays were applied to evaluate the multi-directional differentiation potential of the human PDLSCs. For osteogenic differentiation, 1 × 10^5^ cells were cultured in a 24 well plate until they reached 80% confluence. Then, they were cultured with osteogenic medium. After 28 days, the cells were fixed with 4% paraformaldehyde and stained with 2% Alizarin Red (pH 4.2). PBS washed three times, and calcium nodules stained were identified under a microscope. As to adipogenic differentiation, cells with 100% confluence changed with adipogenic medium A and were incubated for two days. Then, the medium was replaced by adipogenic medium B (standard culture medium supplemented with 10 μg/mL insulin) and incubated for one day. Adipogenic medium A and adipogenic medium B were used in turns as above. Three weeks later, the cells were fixed with 4% paraformaldehyde and stained with 0.3% oil red O for 15 min. PBS washed three times and lipid droplets were observed microscopically.

### 4.5. *In Vitro* Cytocompatibility

The electrospun nanofiber membranes containing zein, zein/gelatin-1, zein/gelatin-2, and zein/gelatin-3 were cut into 10 × 10 mm^2^ samples for a 24 well plate and 5 × 5 mm^2^ specimens for a 96 well plate, sterilized using UV light for 1 h, then put into tissue culture plates. After that, all samples were pre-soaked overnight in the basal medium before cell seeding. The human PDLSCs were seeded with a density of 1 × 10^4^ cells/cm^2^ and incubated at 37 °C and 5% CO^2^, and the medium was changed every 2–3 days.

For the cell proliferation assay, on day 1, day 3, and day 5, the medium was changed to 100 μL serum-free medium; then, 20 μL of MTS solution were added into each well and incubated for 3 h in 37 °C. After that, 100 μL of liquid were taken from each well and the optical density (OD) value was measured using a spectrophotometer at 490 nm. The proliferation assay was assessed by the OD values.

The in vitro adhesion and spreading of human PDLSCs to the fabricated scaffolds were investigated by fluorescence staining. On day 1, day 3, and day 5, the cytoskeleton was stained with vimentin and the nuclei were stained with Hochest 33342. Images were taken with a fluorescence microscope. On day 6, the samples were fixed with a 2.5% glutaraldehyde solution. Then, they were dehydrated with 100% ethanol and sputter-coated with a gold layer. Their cellular adhesion and morphology were obtained using SEM.

To evaluate whether zein/gelatin nanofibrous membranes could provide an environment for human PDLSCs differentiation, ALP activity was determined with an alkaline phosphatase assay kit (Beyotime, Biotechnology, Shanghai, China) using a colorimetric method. After 7 and 14 days, the cells were lysed and collected in a 96 well plate. Next, substrates were added consecutively according to the manufacturer’s method. Finally, the absorption at 410 nm was measured with a spectrophotometer.

### 4.6. Statistical Analysis

The quantitative statistics analysis was performed by one-way ANOVA using the software SPSS 19.0. A single asterisk (*) and double asterisks (**) represent a statistical significance of *p* < 0.05 and *p* < 0.01, respectively.

## 5. Conclusions

In this paper, zein/gelatin cytocompatible nanofiber membranes were successfully fabricated by co-electrospinning technique using HFIP as solvent. Surface morphology, average diameter, contact angle, and mechanical performance as well as cell adhesive capacity in vitro can be controlled by adjusting the amount of gelatin to zein. The zein component resulted in better electrospinnability, while the combination of gelatin was able to enhance the mechanical property, hydrophilicity and cell adhesive ability of the zein membranes. The addition of gelatin to zein extensively changes its hydrophilicity and cytocompatibility, which would provide a more favorable environment for basic cellular adhesion and clinical usage. This study indicates that the electrospun zein/gelatin nanofibrous membranes could be a promising biomaterial for tissue engineering worth further investigation. Next, we plan to investigate a new biomaterial with the ability to promote bone formation based on zein/gelatin electrospun fibers.

## Figures and Tables

**Table 1:** Mechanical properties of electrospun zein/gelatin nanofibers with different compositions.

Sample	Elongation at Break (%)	Tensile Strength (MPa)	Elastic Modulus (MPa)
zein	4.98 ± 0.36	0.79 ± 0.12	35.31 ± 5.53
zein/gelatin-1	4.45 ± 0.35	1.32 ± 0.29	63.18 ± 9.04
zein/gelatin-2	4.60 ± 0.09	1.79 ± 0.16	83.43 ± 5.83
zein/gelatin-3	3.02 ± 0.10	1.88 ± 0.22	133.34 ± 11.22

The data were presented as mean ± standard deviation.

## References

[1] Petersen P.E., Ogawa H. (2005). Strengthening the prevention of periodontal disease: The WHO approach. J. Periodontol..

[2] Heitz-Mayfield L.J.A., Lang N.P. (2013). Surgical and nonsurgical periodontal therapy. Learned and unlearned concepts. Periodontol. 2000.

[3] Bottino M.C., Thomas V., Schmidt G., Vohra Y.K., Chu T.G., Kowolik M.J., Janowski G.M. (2012). Recent advances in the development of GTR/GBR membranes for periodontal regeneration—A materials perspective. Dent. Mater..

[4] Zhu W., Liang M. (2014). Periodontal ligament stem cells: Current status, concerns, and future prospects. Stem Cells Int..

[5] Wang Z.S., Feng Z.H., Wu G.F., Bai S.Z., Dong Y., Chen F.M., Zhao Y.M. (2016). The use of platelet-rich fibrin combined with periodontal ligament and jaw bone mesenchymal stem cell sheets for periodontal tissue engineering. Sci. Rep..

[6] Zhang H., Liu S., Zhu B., Xu Q., Ding Y., Jin Y. (2016). Composite cell sheet for periodontal regeneration: Crosstalk between different types of MSCs in cell sheet facilitates complex periodontal-like tissue regeneration. Stem Cell Res. Ther..

[7] Ratajczak J., Hilkens P., Gervois P., Wolfs E., Jacobs R., Lambrichts I., Bronckaers A. (2016). Angiogenic Capacity of Periodontal Ligament Stem Cells Pretreated with Deferoxamine and/or Fibroblast Growth Factor-2. PLoS ONE.

[8] Chen F., Sun H., Lu H., Yu Q. (2012). Stem cell-delivery therapeutics for periodontal tissue regeneration. Biomaterials.

[9] Zhu B., Liu W., Liu Y., Zhao X., Zhang H., Luo Z., Jin Y. (2017). Jawbone microenvironment promotes periodontium regeneration by regulating the function of periodontal ligament stem cells. Sci. Rep. UK.

[10] Chen F.M., Gao L.N., Tian B.M., Zhang X.Y., Zhang Y.J., Dong G.Y., Lu H., Chu Q., Xu J., Yu Y. (2016). Treatment of periodontal intrabony defects using autologous periodontal ligament stem cells: A randomized clinical trial. Stem Cell Res. Ther..

[11] Taubenberger A.V., Bray L.J., Haller B., Shaposhnykov A., Binner M., Freudenberg U., Guck J., Werner C. (2016). 3D extracellular matrix interactions modulate tumour cell growth, invasion and angiogenesis in engineered tumour microenvironments. Acta Biomater..

[12] Kucinska-Lipka J., Gubanska I., Janik H., Sienkiewicz M. (2015). Fabrication of polyurethane and polyurethane based composite fibres by the electrospinning technique for soft tissue engineering of cardiovascular system. Mater. Sci. Eng. C.

[13] Sadat-Shojai M., Khorasani M., Jamshidi A. (2016). A new strategy for fabrication of bone scaffolds using electrospun nano-HAp/PHB fibers and protein hydrogels. Chem. Eng. J..

[14] Zhijiang C., Qin Z., Xianyou S., Yuanpei L. (2017). Zein/Poly(3-hydroxybutyrate-*co*-4-hydroxybutyrate) electrospun blend fiber scaffolds: Preparation, characterization and cytocompatibility. Mater. Sci. Eng. C.

[15] Escamilla-García M., Calderón-Domínguez G., Chanona-Pérez J.J., Farrera-Rebollo R.R., Andraca-Adame J.A., Arzate-Vázquez I., Mendez-Mendez J.V., Moreno-Ruiz L.A. (2013). Physical and structural characterisation of zein and chitosan edible films using nanotechnology tools. Int. J. Biol. Macromol..

[16] Zhang Y., Cui L., Li F., Shi N., Li C., Yu X., Chen Y., Kong W. (2016). Design, fabrication and biomedical applications of zein-based nano/micro-carrier systems. Int. J. Pharm..

[17] He M., Jiang H., Wang R., Xie Y., Zhao C. (2017). Fabrication of metronidazole loaded poly (epsilon-caprolactone)/zein core/shell nanofiber membranes via coaxial electrospinning for guided tissue regeneration. J. Colloid Interface Sci..

[18] Liao N., Joshi M.K., Tiwari A.P., Park C., Kim C.S. (2016). Fabrication, characterization and biomedical application of two-nozzle electrospun polycaprolactone/zein-calcium lactate composite nonwoven mat. J. Mech. Behav. Biomed..

[19] Lin J., Li C., Zhao Y., Hu J., Zhang L. (2012). Co-electrospun Nanofibrous Membranes of Collagen and Zein for Wound Healing. ACS Appl. Mater. Interfaces.

[20] Zhang Y., Cui L., Che X., Zhang H., Shi N., Li C., Chen Y., Kong W. (2015). Zein-based films and their usage for controlled delivery: Origin, classes and current landscape. J. Control. Release.

[21] Bouman J., Belton P., Venema P., van der Linden E., de Vries R., Qi S. (2016). Controlled Release from Zein Matrices: Interplay of Drug Hydrophobicity and pH. Pharm. Res. Dordr..

[22] Podaralla S., Averineni R., Alqahtani M., Perumal O. (2012). Synthesis of Novel Biodegradable Methoxy Poly (ethylene glycol)-Zein Micelles for Effective Delivery of Curcumin. Mol. Pharm..

[23] Zheng R., Duan H., Xue J., Liu Y., Feng B., Zhao S., Zhu Y., Liu Y., He A., Zhang W. (2014). The influence of Gelatin/PCL ratio and 3-D construct shape of electrospun membranes on cartilage regeneration. Biomaterials.

[24] Mehrasa M., Asadollahi M.A., Nasri-Nasrabadi B., Ghaedi K., Salehi H., Dolatshahi-Pirouz A., Arpanaei A. (2016). Incorporation of mesoporous silica nanoparticles into random electrospun PLGA and PLGA/gelatin nanofibrous scaffolds enhances mechanical and cell proliferation properties. Mater. Sci. Eng. C.

[25] Aldana A.A., Abraham G.A. (2017). Current advances in electrospun gelatin-based scaffolds for tissue engineering applications. Int. J. Pharm..

[26] Shankar K.G., Gostynska N., Montesi M., Panseri S., Sprio S., Kon E., Marcacci M., Tampieri A., Sandri M. (2017). Investigation of different cross-linking approaches on 3D gelatin scaffolds for tissue engineering application: A comparative analysis. Int. J. Biol. Macromol..

[27] Zhang Y.Z., Venugopal J., Huang Z.M., Lim C.T., Ramakrishna S. (2006). Crosslinking of the electrospun gelatin nanofibers. Polymer.

[28] Ma Y., Ji Y., Huang G., Ling K., Zhang X., Xu F. (2015). Bioprinting 3D cell-laden hydrogel microarray for screening human periodontal ligament stem cell response to extracellular matrix. Biofabrication.

[29] Tang R., Wei F., Wei L., Wang S., Ding G. (2014). Osteogenic differentiated periodontal ligament stem cells maintain their immunomodulatory capacity. J. Tissue Eng. Regen. Med..

[30] Yang H., Gao L., An Y., Hu C., Jin F., Zhou J., Jin Y., Chen F. (2013). Comparison of mesenchymal stem cells derived from gingival tissue and periodontal ligament in different incubation conditions. Biomaterials.

[31] Wada N., Menicanin D., Shi S., Bartold P.M., Gronthos S. (2009). Immunomodulatory properties of human periodontal ligament stem cells. J. Cell. Physiol..

[32] Kémoun P., Gronthos S., Snead M.L., Rue J., Courtois B., Vaysse F., Salles J., Brunel G. (2011). The role of cell surface markers and enamel matrix derivatives on human periodontal ligament mesenchymal progenitor responses in vitro. Biomaterials.

[33] Dominici M., Le Blanc K., Mueller I., Slaper-Cortenbach I., Marini F.C., Krause D.S., Deans R.J., Keating A., Prockop D.J., Horwitz E.M. (2006). Minimal criteria for defining multipotent mesenchymal stromal cells. The International Society for Cellular Therapy position statement. Cytotherapy.

[34] Lin Y.X., Ding Z.Y., Zhou X.B., Li S.T., Xie D.M., Li Z.Z., Sun G.D. (2015). In Vitro and In Vivo Evaluation of the Developed PLGA/HAp/Zein Scaffolds for Bone-Cartilage Interface Regeneration. Biomed. Environ. Sci..

[35] Miao Y., Yang R., Deng D.Y.B., Zhang L. (2017). Poly(l-lysine) modified zein nanofibrous membranes as efficient scaffold for adhesion, proliferation, and differentiation of neural stem cells. RSC Adv..

[36] Jiang Y., Jiang L., Huang A., Wang X., Li Q., Turng L. (2017). Electrospun polycaprolactone/gelatin composites with enhanced cell-matrix interactions as blood vessel endothelial layer scaffolds. Mater. Sci. Eng. C.

[37] Hu J., Kai D., Ye H., Tian L., Ding X., Ramakrishna S., Loh X.J. (2017). Electrospinning of poly (glycerol sebacate)-based nanofibers for nerve tissue engineering. Mater. Sci. Eng. C.

[38] Lawrence B.J., Madihally S.V. (2014). Cell colonization in degradable 3D porous matrices. Cell Adhes. Migr..

[39] Eichhorn S.J., Sampson W.W. (2005). Statistical geometry of pores and statistics of porous nanofibrous assemblies. J. R. Soc. Interface.

[40] Hasan A., Memic A., Annabi N., Hossain M., Paul A., Dokmeci M.R., Dehghani F., Khademhosseini A. (2014). Electrospun scaffolds for tissue engineering of vascular grafts. Acta Biomater..

[41] Hsia H.C., Nair M.R., Mintz R.C., Corbett S.A. (2011). The Fiber Diameter of Synthetic Bioresorbable Extracellular Matrix Influences Human Fibroblast Morphology and Fibronectin Matrix Assembly. Plast. Reconstr. Surg..

[42] Bean A.C., Tuan R.S. (2015). Fiber diameter and seeding density influence chondrogenic differentiation of mesenchymal stem cells seeded on electrospun poly(ε-caprolactone) scaffolds. Biomed. Mater..

[43] Razavi S., Karbasi S., Morshed M., Zarkesh E.H., Golozar M., Vaezifar S. (2015). Cell Attachment and Proliferation of Human Adipose-Derived Stem Cells on PLGA/Chitosan Electrospun Nano-Biocomposite. Cell J..

[44] Kai D., Jin G., Prabhakaran M.P., Ramakrishna S. (2013). Electrospun synthetic and natural nanofibers for regenerative medicine and stem cells. Biotechnol. J..

[45] Das S., Pati D., Tiwari N., Nisal A., Sen Gupta S. (2012). Synthesis of Silk Fibroin-Glycopolypeptide Conjugates and Their Recognition with Lectin. Biomacromolecules.

[46] Jia J., Duan Y., Yu J., Lu J. (2008). Preparation and immobilization of soluble eggshell membrane protein on the electrospun nanofibers to enhance cell adhesion and growth. J. Biomed. Mater. Res. A.

[47] Zhang Y., Wang Q.S., Yan K., Qi Y., Wang G.F., Cui Y.L. (2016). Preparation, characterization, and evaluation of genipin crosslinked chitosan/gelatin three-dimensional scaffolds for liver tissue engineering applications. J. Biomed. Mater. Res. A.

[48] Gautam S., Dinda A.K., Mishra N.C. (2013). Fabrication and characterization of PCL/gelatin composite nanofibrous scaffold for tissue engineering applications by electrospinning method. Mater. Sci. Eng. C.

[49] Ghasemi-Mobarakeh L., Prabhakaran M.P., Morshed M., Nasr-Esfahani M.H., Ramakrishna S. (2008). Electrospun poly(3-caprolactone)/gelatin nanofibrous scaffolds for nerve tissue engineering. Biomaterials.

